# Derecho a la salud en Colombia: cumplimiento de órdenes de la Corte Constitucional al año 2024

**DOI:** 10.15446/rsap.V27n4.121060

**Published:** 2025-07-01

**Authors:** Jairo H. Restrepo-Zea, Lesny D. Palacios-Romaña

**Affiliations:** 1 JR: Econ. M. Sc. Políticas Públicas. Coordinador Grupo de Economía de la Salud (GES). Facultad de Ciencias Económicas, Universidad de Antioquia. Medellín, Colombia. jairo.restrepo@udea.edu.co Universidad de Antioquia Facultad de Ciencias Económicas Universidad de Antioquia Medellín Colombia jairo.restrepo@udea.edu.co; 2 LP: Adm. Salud con énfasis en Servicios de Salud. M. Sc. Administración en Salud, M. Sc. Finanzas. Grupo de Economía de la Salud (GES). Facultad de Ciencias Económicas, Universidad de Antioquia. Medellín, Colombia. lesny.alacios@udea.edu.co Universidad de Antioquia Facultad de Ciencias Económicas Universidad de Antioquia Medellín Colombia lesny.alacios@udea.edu.co

**Keywords:** Derecho a la salud, Colombia, cobertura universal en salud, financiación de la salud, política pública, jurisprudencia *(fuente: DeCS, BIREME)*, Right to health, Colombia, universal health coverage, healthcare financing, public policy, jurisprudence *(source: MeSH, NLM)*

## Abstract

En julio de 2008, la Corte Constitucional de Colombia emitió la sentencia T-760, mediante la cual otorgó a la salud el carácter de derecho humano fundamental autónomo. En la sentencia, motivada por el elevado número de acciones de tutela o amparo que solicitaban a los jueces proteger el derecho a la salud, la Corte señaló fallas estructurales que impedían el goce efectivo del derecho a la salud. En respuesta a esta problemática, la Corte impartió una serie de órdenes dirigidas a los actores del sistema de salud y creó una sala especial de seguimiento a dichas órdenes. Este trabajo tiene como objetivo identificar los avances en la implementación de las órdenes de la Corte y los cambios en el sistema en torno al plan de beneficios, la cobertura y el acceso, la sostenibilidad financiera y el flujo de los recursos. Los resultados indican que al año 2024 no se habían registrado avances significativos, con valoraciones similares a las de 2015 y 2018. Aunque se han identificado ciertos progresos, estos no alcanzan niveles altos de cumplimiento, y persisten obstáculos en el acceso a servicios y en el flujo de recursos. Se sugiere realizar análisis que den cuenta de la incidencia del cumplimiento de las órdenes en la garantía del derecho a la salud.

## Abstract (en)

In July 2008, the Constitutional Court of Colombia issued judgment T-760, by which It granted health the status of an autonomous fundamental human right. In the ruling, motivated by the high number of tutela or amparo actions that asked judges to protect the right to health, the Court pointed out structural flaws that prevented the effective enjoyment of the right to health. In response to this problem, the Court issued a series of orders addressed to the actors of the health system and created a special chamber to follow up on these orders. This work aims to identify progress in the implementation of Court orders and changes in the system around benefit plan, coverage and access, financial sustainability, and resource flow. The results indicate that no significant progress has been made by 2024, with assessments similar to those of 2015 and 2018. Although some progress has been identified, it does not reach high levels of compliance, and obstacles persist in access to services and in the flow of resources. It is suggested that analyses be carried out that account for the impact of compliance with the orders on the guarantee of the right to health.

La Constitución Política de Colombia establece que la atención en salud es un servicio público a cargo del Estado (Art. 49), y consagra la obligatoriedad de la seguridad social con la dirección del Estado, regida por los principios de eficiencia, universalidad y solidaridad (Art. 48) 1. La materialización de estos preceptos fue reglamentada mediante la Ley 100 de 1993, con la cual se creó el Sistema General de Seguridad Social en Salud (SGSSS). Esta reforma se destacó por adoptar un enfoque integral y universal, a diferencia de la mayoría de las reformas en América Latina, que para entonces se habían limitado a beneficiar a grupos específicos de la población, especialmente a las clases medias urbanas 2. La gran aspiración que traía la Ley 100 era la de cubrir a toda la población con un conjunto amplio de servicios y medicamentos, el denominado Plan Obligatorio de Salud (POS), hoy conocido como Plan de Beneficios en Salud (PBS), y de este modo, pasar de una cobertura del seguro de salud que apenas rondaba el 25% de la población en 1992, al 100% al concluir el año 2000.

La implementación de la Ley 100 vino acompañada de un aumento significativo de los recursos destinados al sistema de salud, en forma consistente con el propósito de la cobertura universal. Se destaca el aumento de la cotización, que pasó del 7% del ingreso en 1993 al 12% a partir de 1995, y mayores recursos fiscales a partir de la destinación a salud del 25% de las transferencias territoriales, junto con la asignación de recursos del presupuesto general de la nación, lo que en conjunto pudo significar un aumento del gasto total en salud del orden del 1% del Producto Interno Bruto (PIB) 3.

Sin embargo, para el año 2000, la cobertura del sistema apenas alcanzaba el 58,5% de la población nacional. Adicionalmente, aunque la Ley 100 contemplaba el POS para la población afiliada, y si bien la reglamentación de este plan dejó vacíos normativos que llevaron a unas "zonas grises", al no quedar claro si determinados procedimientos, medicamentos o servicios estaban o no cubiertos y, por tanto, si o no serían responsabilidad de las entidades promotoras de salud (EPS), la propia ley dejó planteada la posibilidad de demandar atenciones que no estuvieran incluidas en el POS y fueran tramitadas mediante el mecanismo de comité técnico científico a cargo de cada EPS, y el valor correspondiente fuera recobrado al Fondo de Solidaridad y Garantía (Fosyga) , hoy Administradora de los Recursos de la Salud (ADRES) 4. Fue así como se dio lugar a un incremento sostenido de los recobros por parte de las EPS 5, lo que a la postre derivaría en deudas del Estado con las EPS, generando problemas en el adecuado flujo de recursos dentro del sistema 6.

Ahora bien, a la par con los avances en la cobertura y la expectativa de cubrir a toda la población, sumado a la adopción de la tutela como mecanismo de amparo para proteger los derechos fundamentales, también derivado de la Constitución de 1991, en el país comenzó a registrarse un alto número de tutelas por las cuales los ciudadanos pedían a los jueces ordenar el acceso a servicios de salud, bien porque fueran negados a pesar de estar incluidos en el POS, o porque se trataba de prestaciones que estaban por fuera de este. Entre 1999 y 2008 se interpusieron 674 612 tutelas relacionadas con el derecho a la salud, con un aumento en la tasa de 5,36 tutelas por cada 10 000 afiliados en 1999, a 32,16 por cada 10 000 afiliados en 2008. De estas acciones, el 54,4% correspondía a la exigencia de bienes y servicios que sí estaban incluidos en el POS 6.

En cumplimiento de su función de defensa de los derechos y del control de constitucionalidad de las leyes (Arts. 241,242,2043) 1, en 2008, la Corte Constitucional se pronunció mediante la Sentencia T-760 con el objetivo de garantizar el acceso a los servicios de salud, mejorar el funcionamiento del sistema, reducir el número de demandas judiciales y declarar la salud como derecho fundamental autónomo. En esta sentencia, la Corte recogió 22 tutelas, de las cuales 20 fueron interpuestas por ciudadanos a quienes se había vulnerado el derecho a la salud, y dos presentadas por la EPS Sanitas contra el Consejo Superior de la Judicatura y el Ministerio de la Protección Social para solicitar claridad respecto a las reglas de recobro ante el Fosyga 7.

Además de resolver las 22 tutelas, la Corte impartió 35 órdenes, de las cuales 16 fueron de carácter general y dirigidas principalmente al Gobierno nacional, con el fin de corregir las fallas estructurales identificadas en el sistema de salud. Las órdenes se agrupan en seis ejes temáticos: I.) precisión, actualización, unificación y acceso a planes de beneficios; II.) sostenibilidad financiera y flujo de recursos; III.) cobertura universal y sostenible de los servicios; IV.) medición del número de las tutelas; V.) reglamentación de las cartas de derechos y deberes de los usuarios, y de desempeño de las EPS; y VI.) difusión de la sentencia entre los funcionarios judiciales 8. Estas órdenes generales se alinean con el modelo establecido por la Ley 100 y sus reformas posteriores, ya que la Corte consideró que dicha ley ofrecía un marco normativo adecuado para garantizar el derecho a la salud. El problema principal, según el alto tribunal, no radicaba en la ley misma, sino en su deficiente implementación y en el incumplimiento de lo establecido en ella 7,9.

Adicionalmente, la Sentencia T-760 señaló que los indicadores de gestión y de resultados en el ámbito de la salud, anteriormente establecidos en el artículo 2 de la Ley 1122 de 2007, debían incorporar la medición del goce efectivo del derecho a la salud. En la misma línea, la Ley Estatutaria de Salud 10, que regula este derecho fundamental, establece mecanismos para su protección y obliga al gobierno a implementar un sistema de indicadores que evalúen el goce efectivo del derecho a la salud. Esta medición debe considerar los elementos de accesibilidad, disponibilidad, aceptabilidad y calidad de los servicios de salud.

Para el año 2018, una década después de la promulgación de la sentencia, el seguimiento realizado por la Corte Constitucional, así como diversos estudios 7,8,11,12, evidenciaron que aún no se había alcanzado un cumplimiento pleno de las órdenes impartidas. En particular, el análisis realizado por el Grupo de Economía de la Salud (GES) 8 destacó que, si bien se registraron avances importantes en aspectos como la actualización periódica y unificación del POS para toda la población, el incremento del valor de la unidad de pago por capitación (UPC) del régimen subsidiado y el pago de recobros y el control de precios de medicamentos, persistían serias dificultades en términos de acceso efectivo y equidad en la atención en salud 11.

Este artículo tiene como propósito analizar el grado de cumplimiento de las órdenes impartidas por la Corte Constitucional en la Sentencia T-760 de 2008, con especial énfasis en el periodo comprendido entre 2018 y 2024, retomando el análisis realizado por el GES 8 tras cumplirse una década de la sentencia. Para ello, se examinan los autos de seguimiento emitidos por la Corte y las principales medidas adoptadas para dar cumplimiento a lo ordenado. Asimismo, se busca aportar un insumo académico y de debate en el ámbito de las políticas públicas, que permita reflexionar e indagar sobre los efectos de dichas órdenes en el funcionamiento del sistema de salud y su incidencia en el goce efectivo del derecho a la salud.

## Avances de la Sentencia T-760

La Sala Especial de Seguimiento de la Sentencia T-760 de la Corte Constitucional realiza valoraciones para verificar el nivel de cumplimiento de las órdenes impartidas a partir de 2008. En la Tabla 1 se presentan los resultados de estas valoraciones correspondientes a los periodos 2015-2018 y 2021-2024, con el propósito de comparar los avances alcanzados desde las últimas valoraciones realizadas por el GES. La calificación consiste en cinco niveles de cumplimiento, a partir de los cuales la Sala puede emitir nuevas órdenes complementarias, mantener el seguimiento o, cuando se considere que se ha cumplido satisfactoriamente, declarar el cumplimiento definitivo de la orden 12. 

**Tabla 1:** Colombia: Seguimiento de órdenes de la Sentencia T-760 de 2008, Corte Constitucional.

Precisión, actualización, unificación y acceso a planes de beneficios					
Actualización integral y periódica del PBS	17, 18	Medio	Medio	**Figure:** 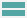	410 de 2016 010 de 2024
Crear un registro de servicios negados alimentado por la información de las EPS	19	Bajo	Bajo	**Figure:** 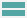	411 de 2015 005 de 2024
Crear un *ranking* de EPS e IPS	20	Bajo para IPS Medio para EPS	Bajo para IPS Medio para EPS	**Figure:** 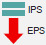	591 de 2016 708 de 2024 1089 de 2022
Unificación del PBS	21, 22	Medio	Medio	**Figure:** 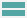	411 de 2016 996 de 2023
Respecto del componente de suficiencia de presupuestos máximos	21, 22	Bajo**	Incumplimiento general	**Figure:** 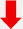	996 de 2023 2049 de 2024
Creación de un mecanismo directo para autorizar servicios no cubiertos por el PBS	23	Bajo	Medio en prescripción de servicios, capacitación a prescriptores y autonomía médica. Bajo desempeño de JPS***, inclusión medicamentos en Unirs****	**Figure:** 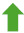	001 de 2017 2566 de 2023
Sostenibilidad financiera y flujo de recursos					
Asegurar el flujo de recursos al interior del sistema y su sostenibilidad financiera	24	Incumpliendo parcial	Bajo	**Figure:** 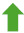	263 de 2012 2882 de 2023
Eliminar las causales de glosas denominadas “fallo de tutela” y “principio activo POS” dando trámite al pago de los recobros represados a septiembre de 2008	25, 26	General	General	**Figure:** 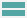	186 de 2018 112 de 2016
Rediseñar el procedimiento de recobro	27	Bajo	Bajo	**Figure:** 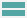	071 de 2016 1299 de 2024
Cobertura y acceso					
Asegurar la cobertura universal	29	Incumpliendo parcial	Medio afiliación	**Figure:** 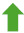	314 de 2016 496 de 2022 607 de 2024
Medir el número de las tutelas					
Medición de acciones de tutela	30	Bajo	Medio	**Figure:** 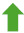	590 de 2016 1680 de 2022

(*) No se aplica la valoración de cumplimiento para las órdenes 1 a 16, ya que están orientadas a la resolución de casos individuales, en los cuales se estableció un cumplimiento de carácter inmediato.

(**) Esta orden se evaluó en los periodos 2023 y 2024, dado que los presupuestos máximos nacen en el 2019.

(***) Junta de Profesionales de la Salud.

(****) Usos no incluidos en el registro sanitario.

Fuente: Sala Especial de Seguimiento de la Sentencia T-760 de la Corte Constitucional.

La escala de valoración tiene las siguientes denominaciones: incumplimiento general, cuando no se adoptan medidas para superar la falla; cumplimiento bajo, en la medida en que si bien se adoptan medidas, estas resultan insuficientes o solo atienden al aspecto formal de la orden sin garantizar la protección del derecho; cumplimiento medio, cuando se implementan medidas y se reportan avances, pero estos son parciales; cumplimiento alto, si se han tomado medidas adecuadas con resultados significativos y sostenibles, pero es necesario mantener la supervisión, y cumplimiento general, cuando las medidas son adecuadas y los resultados demuestran que se ha superado la falla estructural.

Como se observa en la Tabla 1, al analizar el cumplimiento de las órdenes se observa un nivel de cumplimiento medio a bajo, con algunos avances puntuales y retrocesos significativos. Entre los avances más destacados se encuentran los relacionados con las órdenes 25 y 26, que tratan sobre la eliminación de los motivos de glosa como "principio activo en el POS" y "fallo de tutela", así como el pago de los recobros atrasados al 30 de septiembre de 2008. En estos casos, se ha logrado un cumplimiento general.

También se reportan progresos en órdenes relacionadas con la creación de un mecanismo directo para autorizar servicios no cubiertos por el PBS, el aseguramiento del flujo de recursos dentro del sistema, la sostenibilidad financiera, la cobertura universal y la medición de las acciones de tutela. No obstante, en algunos de estos aspectos, el avance ha sido apenas incipiente. Por otro lado, existen órdenes que mantienen una valoración media, como aquellas vinculadas a la unificación y actualización del PBS, debido a la falta de medidas permanentes que garanticen su cumplimiento. Finalmente, se destacan las órdenes cuyo cumplimiento permanece en niveles bajos o ha retrocedido, especialmente aquellas que afectan directamente la sostenibilidad financiera del sistema y el flujo de recursos, y en los últimos años tiende a empeorar dicho nivel de cumplimiento. Asimismo, las órdenes tendientes a identificación y seguimiento de instituciones que niegan los servicios han permanecido en un cumplimiento bajo.

## El plan de beneficios en salud (PBS)

La unificación de los planes de beneficios, entre la población contributiva y la subsidiada inició el 1.o de octubre del 2009 mediante el Acuerdo 04 de la Comisión de Regulación en Salud (CRES). Este proceso comenzó con la inclusión de los menores de edad y se extendió progresivamente a los demás grupos etarios, completándose en julio de 2012. Esta medida respondió a la necesidad de corregir la desigualdad en las coberturas entre ambos regímenes, lo que vulneraba especialmente el derecho de los afiliados al régimen subsidiado. A partir de entonces, con ocasión de la Ley Estatutaria de Salud (Ley 1751 de 2015), se acogió el término de PBS, sustituyendo conceptualmente al POS, en la medida en que este era un plan explícito y se ha dado lugar a un plan implícito. Además, con la actualización realizada en 2021 13, Colombia logró avanzar hacia un enfoque de plan integral de salud, incorporando el 97% de los procedimientos médicos autorizados en el país, con logros significativos y destacados en materia de cobertura universal 14,15.

No obstante, la garantía efectiva de este plan depende de que los recursos financieros sean suficientes, especialmente la UPC. Esta corresponde a la prima anual que las EPS reciben por cada afiliado al sistema para cubrir los costos predecibles asociados a la prestación de los servicios médicos 16. De manera que la UPC debe ser suficiente para garantizar el cubrimiento de todos los tratamientos y tecnologías que no estén expresamente excluidos del PBS. Sobre el particular, varios análisis y el reclamo reiterado de las EPS dan cuenta de una UPC insuficiente 17,18. Además, según lo ha señalado la Corte Constitucional, el cálculo de la UPC en las últimas vigencias ha tenido un impacto grave y significativo en la sostenibilidad financiera del SGSSS 17. Esto ha llevado a que la Corte ordene al MSPS la creación y el seguimiento de una mesa de trabajo con el objetivo de revisar la suficiencia de la UPC para el año 2024 y ajustar la metodología utilizada para su cálculo en futuras vigencias. Asimismo, se ha exigido al MSPS que presente evidencia del diálogo sostenido con las EPS sobre el valor de la UPC, con base en los datos utilizados y los resultados obtenidos.

Por otra parte, a pesar de la actualización periódica del PBS, persisten "zonas grises" en las coberturas. Estas se derivan del mecanismo de listado de exclusiones implementado por el Ministerio, que contempla un listado taxativo de servicios financiados mediante la UPC y otro de exclusiones. Sin embargo, existen procedimientos y medicamentos que no figuran en ninguno de los dos, lo que genera ambigüedad normativa e incertidumbre tanto para los prestadores como para los usuarios del sistema. Esta situación ha contribuido a que se mantenga un uso recurrente de la acción de tutela como mecanismo para garantizar el derecho fundamental a la salud 19,20.

La creación de un mecanismo para autorizar servicios no incluidos en el PBS, a través de la herramienta Mi Prescripción (MIPRES), también ha representado un avance importante en el cumplimiento de las órdenes de la Corte Constitucional. Esta plataforma ha permitido ejercer un mayor control y seguimiento sobre el gasto en servicios y tecnologías no financiados con recursos de la UPC 15. No obstante, esta herramienta presenta limitaciones que afectan la oportunidad y continuidad en el acceso a los servicios de salud. Entre estas se encuentra el desconocimiento de la ruta para acceder a los servicios prescritos, problemas de conectividad que afectan la continuidad del tratamiento, errores en el diligenciamiento de la plataforma, dificultades con la homologación de códigos, ausencia de ciertas tecnologías en el sistema, falta de capacitación a los profesionales de la salud, no entrega de los medicamentos formulados y errores en las cantidades prescritas. De igual forma, se siguen presentado tutelas para acceder a la prescripción de servicios no financiados con la UPC y quejas relacionados con esta herramienta 21.

Adicionalmente, se implementó el mecanismo de presupuestos máximos, que constituyen una asignación adicional de recursos que se determina cada año con el fin de financiar servicios y tecnologías que no están incluidos en la UPC. Esto se debe a que presentan condiciones inciertas, una alta variabilidad en sus precios o corresponden a servicios sociales complementarios ordenados por decisión judicial 22. Sin embargo, esta estrategia ha resultado insuficiente para evitar la acumulación de recobros por parte de las EPS, lo que ha continuado afectando el flujo de recursos en el sistema. Estos aspectos son analizados con mayor detalle en secciones posteriores.

## Cobertura y acceso

Colombia ha logrado avances significativos en materia de cobertura en salud, alcanzando en 2023 una afiliación del 98,93% de la población al sistema de salud y los regímenes de excepción 23 (Tabla 2). Este indicador refleja un progreso notable en términos de aseguramiento formal. Sin embargo, alcanzar una cobertura casi universal no garantiza por sí solo el acceso efectivo y equitativo a los servicios de salud. Aún persisten desafíos importantes para lograr el acceso de toda la población, especialmente población rural dispersa, que enfrenta mayores barreras. En términos de acceso, se evidencian desigualdades marcadas entre los grandes centros urbanos y las zonas rurales o geográficamente aisladas. En estas últimas, los problemas de infraestructura, la limitada oferta de servicios y la escasa disponibilidad de personal de salud capacitado continúan siendo obstáculos estructurales que restringen la atención oportuna y de calidad 20,24. Estas brechas territoriales no solo afectan la eficiencia del sistema, sino que perpetúan inequidades que contradicen el principio de equidad en el acceso a la salud.

**Tabla 2:** Colombia: Indicadores del sistema de salud 1996-2023

Indicadores	1996	2000	2008	2017	2023
Cobertura y afiliación por régimen					
Población proyectada a	37 472 184	40 295 563	44 451 147	49 291 609	52 422 921
Cobertura de aseguramiento	39%	58,50%	83,30%	94,40%	98,93%
Afiliación régimen contributivo b	14 618 732	14 051 804	18 405 579	22 045 454	23 527 968
Afiliación régimen subsidiado b	5 981 778	9 510 488	18 603 005	22 434 577	26 174 133
Acceso a los servicios de salud. Barreras al acceso d					
% personas que no acceden a servicios	24,30%b	25%	21,50%	21%	33,90%
% no acceden por falta de dinero	44,20%b	29,50%	23,40%	5,10%	4,90%
		Tutelas			
N.o de tutelas salud	Nd	24 843	142 957	197 655	197 719
Tutelas en salud por 10 000 afiliados	Nd	11,0	35,0	42,3	38,1
Financiación					
Recobros ($ billones) e	$0	0$	$2,2	$3,1	$0,87
UPC-S/UPC-C	62,00%	53,00%	56,00%	89,00%	87,00%
UPC-C real ($ de 2023) f	687 153	773 988	777 284	970 122	1 289 246
UPC- S real ($ de 2023) f	425 922	412 081	437 620	867 892	1 121 396

Fuente: ^a^ DANE. Proyecciones de población; ^b^ Ministerio de Salud, datos sobre población afiliada; ^c^ Defensoría del Pueblo, información sobre tutelas; ^d^ DANE. Encuesta Nacional de Calidad de Vida, 1997, 2003, 2008 y 2017,2023; ^e^ Ministerio de Salud, Informe anual; ^f^ CNSSS, Acuerdo 322 de 2000 y Acuerdo 161 de 2000; Ministerio de Salud, Acuerdo 379 de 2008, Resolución 6411 de 2016, Resolución 2809 de 2022.

## Sostenibilidad financiera y flujo de recurso

**Figura 1:**
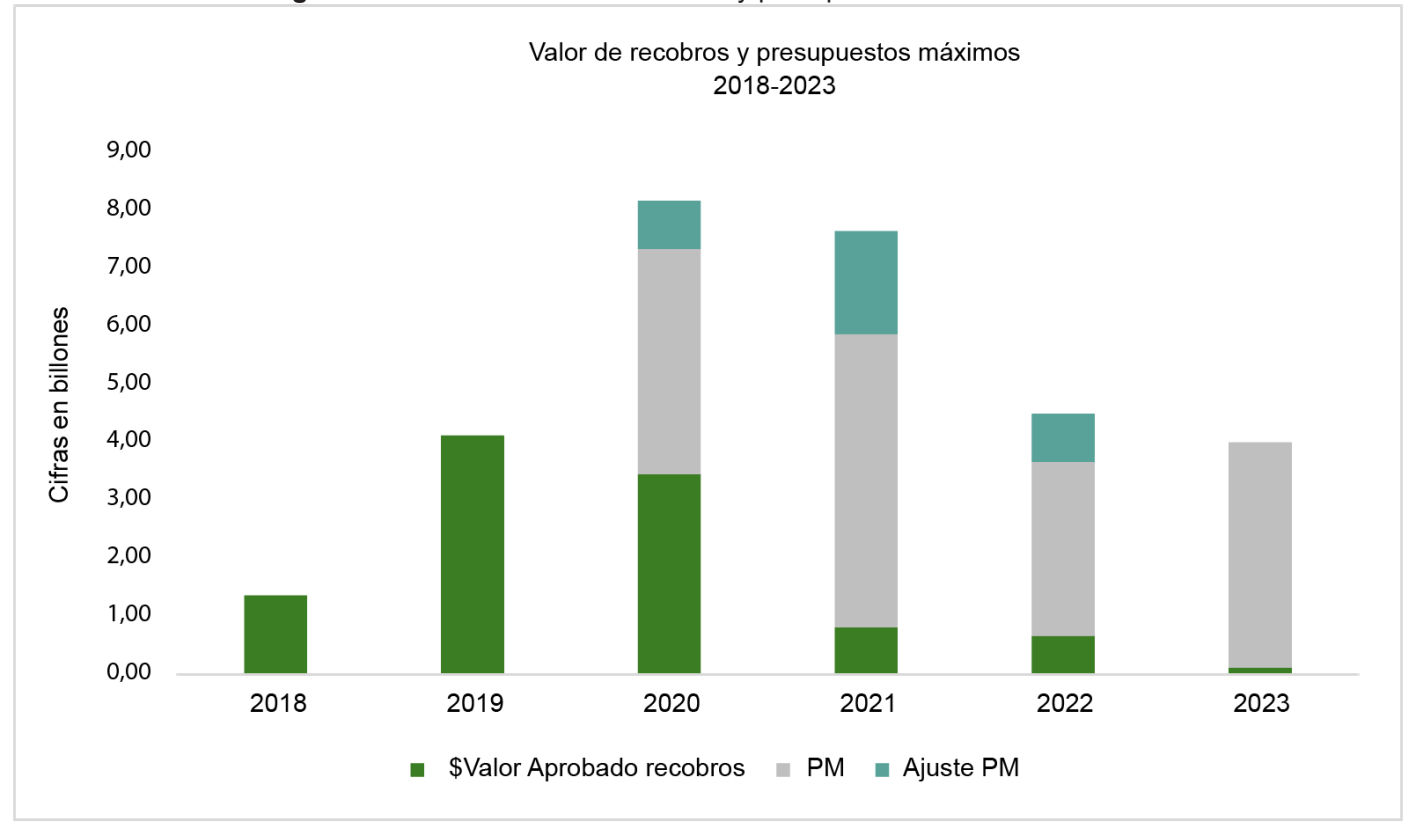
Colombia. Valor de recobros y presupuestos máximos 2018-2023

Las órdenes de la Corte Constitucional en materia de financiación han buscado, además de garantizar los recursos suficientes mediante la UPC, agilizar el procedimiento de recobros ante el entonces Fosyga y garantizar un flujo oportuno y suficiente de todos los recursos. Sobre el particular, el sistema enfrenta el mismo flagelo, representado en un flujo de recursos inadecuado y la vigencia de los recobros cuando se esperaba que fueran eliminados (Figura 1), lo que afecta la sostenibilidad financiera y limita la capacidad de respuesta ante las necesidades de la población.

*PM: presupuestos máximos. Fuente: Respuesta del ADRES a la Comisión Séptima Cámara de Representantes, cálculos propios.

En 2019 se diseñaron los presupuestos máximos para financiar la prestación de servicios y tecnologías en salud no cubiertos con la UPC, mediante el artículo 240 de la Ley 1955 25. Estos recursos son entregados de forma anticipada a las EPS con el fin de garantizar la atención de servicios excluidos del PBS y evitar los recobros. No obstante, la Corte Constitucional ha señalado problemas de inoportunidad en el flujo de recursos, atribuibles a dificultades en los procesos de verificación, control y pago por parte de la ADRES. Asimismo, como se observa en la Figura 1, los recobros se mantienen vigentes para cubrir nuevos servicios y tecnologías que no son financiados ni con la UPC ni con los presupuestos máximos.

En las valoraciones más recientes, la Corte no ha podido constatar la suficiencia ni de la UPC ni de los presupuestos máximos 18, debido a la deficiencia de los sistemas de información disponibles. Aunque la UPC del régimen subsidiado (UPC-S) se calcula con datos propios de este régimen, la Corte considera que el MSPS no ha demostrado que dicha información sea técnica y confiable para establecer con precisión esta prima. Además, no se ha logrado equiparar el porcentaje del valor de la prima del régimen subsidiado al 95 % del monto establecido para la UPC del régimen contributivo (UPC-C), tal como lo exige la normatividad vigente (el Auto 411 de 2016 de la Corte Constitucional).

En relación con los presupuestos máximos, se identificó que las EPS enfrentan una siniestralidad superior a la prevista, lo que genera déficit en dichos presupuestos. La Sala Especial de Seguimiento detectó problemas en la fijación y los reajustes de los presupuestos, así como retrasos en los pagos. Además, evidenció deficiencias en la expedición de la metodología requerida para su elaboración oportuna, debido a demoras en la entrega y recolección de información completa necesaria para su análisis y cálculo.

El mecanismo del giro directo fue diseñado para garantizar un flujo oportuno de recursos en el sistema; sin embargo, la Sala ha señaló falencias en su aplicación. Aunque esta medida contribuye a mejorar la oportunidad en la disponibilidad de recursos, su impacto es limitado y no representa un avance significativo para superar esta falla estructural.

Por otra parte, existe una deuda creciente 26 y una brecha considerable entre la deuda que las EPS reconocen tener pendiente por pagar y la que las IPS estiman estar esperando cobrar 27. Esta discrepancia está vinculada a las deficiencias del sistema de información, que no permiten mantener una trazabilidad adecuada de la facturación presentada por los prestadores de servicios de salud, dificultando así la determinación precisa del estado real de la deuda.

## Goce efectivo al derecho a la salud

El concepto del goce efectivo del derecho está consagrado en artículo 2 de la Constitución Política colombiana, donde se establece como fin esencial del Estado garantizar la efectividad de los principios, derechos y deberes consagrados en la Carta Magna. En consonancia con este propósito, la Constitución también establece la acción de tutela como un mecanismo mediante el cual la ciudadanía puede reclamar ante los jueces la protección inmediata de sus derechos fundamentales, en casos de vulneración o amenaza por acción u omisión de cualquier autoridad (Art. 86) 1. Por ello, el número y la naturaleza de las tutelas presentadas se utilizan como un indicador relevante para medir la vulneración o garantía del goce efectivo del derecho a la salud.

**Figura 2:**
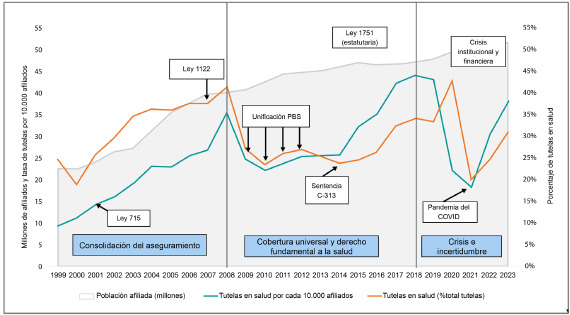
Colombia: tutelas en salud, 1999-2023 (% total tutelas y tasa por cada 10 000 afiliados)

En la Figura 2 se observa que tras la implementación de las medidas orientadas al cumplimiento de las órdenes establecidas en la Sentencia T-760/ 08, el número de tutelas en salud se redujo inicialmente. Sin embargo, a partir de 2015 se presenta una tendencia creciente en el número de tutelas, superando incluso los niveles registrados antes de la sentencia. Los picos más altos se registraron en 2018, con 44 tutelas por cada 10 000 afiliados, y en 2020, cuando las tutelas en salud representaron el 43% del total de tutelas en el país. La única disminución significativa se dio durante la pandemia de COVID-19, debido seguramente a las medidas de confinamiento, tras lo cual la tendencia al aumento continuó hasta el último año analizado.

Fuente: Informe de tutelas y el derecho a la salud y la seguridad social de la Defensoría del Pueblo, cálculos y elaboración del GES.

De acuerdo con las valoraciones de la Corte Constitucional y el comportamiento del sector, se han identificado varios factores que parecen explicar el aumento en las tutelas: i) La Ley Estatutaria 1751 de 2015 relaja los límites y se amplía el reclamo por el derecho; ii) aumento de los beneficios en salud y iii) el deterioro del sistema.

La Ley Estatutaria 1751 de 2015 regula el derecho fundamental a la salud, con el objetivo de resolver problemas relacionados con la calidad, la oportunidad y el acceso a los servicios de salud. Esta normativa amplía los beneficios del sistema, estableciendo el plan de beneficios basado en un listado de exclusiones, en lugar de un listado cerrado de servicios. Esta característica puede haber motivado a la población a exigir tecnologías y medicamentos que se encuentran en las denominadas "zonas grises" del sistema, es decir, aquellos servicios que no están explícitamente incluidos ni excluidos en el plan, generando mayor litigiosidad y solicitudes judiciales para garantizar su acceso.

Como se mencionó anteriormente en relación con los avances en el PBS, en 2022 se cubría el 97% de los procedimientos médicos autorizados en el país. En cuanto a medicamentos, el PBS incluía el 89% del total de Códigos Únicos de Medicamento (CUMS), lo que representa más de 35 000 referencias farmacológicas 28. Si bien la ampliación de beneficios constituye un avance significativo en el reconocimiento del derecho a la salud, su implementación efectiva está condicionada a la suficiencia de la UPC. La incertidumbre sobre la suficiencia de la UPC puede llevar a que las EPS incurran en prácticas de negación o dilación en la prestación de servicios, lo que a su vez contribuye al incremento de acciones de tutela interpuestas por los usuarios para acceder a las atenciones requeridas.

El sistema de salud continúa enfrentando serias dificultades relacionadas con el flujo de recursos, las deudas entre los distintos actores y el deterioro financiero, tanto de las entidades promotoras como de los prestadores de servicios. Estas problemáticas han generado un ambiente de inestabilidad que ha desembocado en la liquidación o intervención de varias EPS de gran tamaño, con impactos significativos sobre la población afiliada. Uno de los años más críticos fue 2015, cuando seis EPS fueron liquidadas o puestas bajo intervención, entre ellas Saludcoop y Caprecom, afectando aproximadamente a 9,7 millones de usuarios. De manera similar, en 2019 cinco EPS enfrentaron procesos similares, destacándose los casos de Cafesalud y Saludvida, con una población afectada cercana a los 4,7 millones de afiliados 29,30. Actualmente, la situación es aún más compleja, pues 10 de las 29 EPS que operan en el país se encuentran intervenidas o bajo medida de vigilancia especial. Estas entidades concentran cerca de 29,8 millones de afiliados, lo que representa el 57,09% del total de afiliados al sistema de salud 30. A esta crisis se suma la intención manifestada por varias EPS de cesar sus operaciones, una solicitud que ha sido rechazada por el Gobierno.

También se cuenta con otras causas atribuibles al aumento de las tutelas, como lo plantean Otálvaro et al. 31, para quienes el mayor número de tutelas que siguió a la promulgación de Ley estatutaria está explicado en gran medida por cuatro factores: el aumento de la conciencia jurídica popular frente al derecho a la salud, la interpretación arbitraria de la Ley Estatutaria por los operadores jurídicos, la crisis financiera y administrativa de las EPS, y los fallos del diseño institucional y de la capacidad regulatoria.

## Hacia una medición del goce efectivo del derecho a la salud

De acuerdo con la ONU 32 el derecho a la salud incluye cuatro elementos esenciales e interrelacionados, los cuales son: i) disponibilidad, existencia suficiente de establecimientos, bienes y servicios de salud; ii) accesibilidad, establecimientos, bienes y servicios de salud accesibles para toda la población, sin discriminación alguna, dentro de la jurisdicción del Estado. La accesibilidad tiene cuatro dimensiones: no discriminación, accesibilidad física, accesibilidad económica (asequibilidad) y accesibilidad de la información; iii) aceptabilidad, los prestadores y los servicios de salud deben ser respetuosos de la ética médica y culturalmente apropiados para el contexto local y; iv) calidad, grado en que los servicios de salud incrementan la probabilidad de alcanzar resultados sanitarios deseados y se ajustan a conocimientos profesionales basados en datos probatorios. Por consiguiente, se podría inferir qué, para determinar la materialización del goce efectivo del derecho a la salud, primero es necesario medir el estado de los componentes definidos por la ONU 32 (Tabla 3).

**Tabla 3:** Componentes del derecho a la salud Componente Medición

Componente	Medición
Disponibilidad	▪ Distribución equitativa de los servicios u oferta de servicios ▪ Suficiencia de servicios ▪ Disponibilidad de talento humano mínimo ▪ Dotación mínima
Accesibilidad	Obstáculos físicos, geográficos, económicos, información y de otra índole a los sistemas y los servicios de salud
Aceptabilidad	Satisfacción Respeto y protección del ejercicio de prácticas medicinales
Calidad	Seguridad Eficacia Oportunidad Centrados en la persona Equidad Integridad Eficiencia

Fuente: ONU. Observación General N.o 14 del Comité de Derechos Económicos, Sociales y Culturales.

Desde la promulgación de la Sentencia 760, se ha buscado garantizar la materialización y el goce efectivo del derecho a la salud. En este sentido, la Corte Constitucional estableció que el MSPS debía desarrollar indicadores específicos para evaluar el desempeño del sistema de salud desde la perspectiva del goce efectivo de este derecho.

Posteriormente, mediante el Auto 590 de 2016, la Corte ordenó al MSPS la creación de indicadores de goce efectivo como herramienta fundamental para evaluar la gestión de los diferentes actores del sistema. Esta directriz reiteró que los indicadores de gestión y de resultados definidos en el artículo 2 de la Ley 1122 de 2007 debían incorporar explícitamente la medición del goce efectivo del derecho a la salud.

La Corte ratificó y amplió esta orden a través del Auto A077A de 2020, en el que instruyó al MSPS a fortalecer y ampliar dichos indicadores, integrando además los determinantes sociales de la salud. Asimismo, se estableció que estos indicadores debían ser aplicables a todos los niveles y actores del sistema, incluyendo las EPS, las IPS, las entidades territoriales y demás agentes involucrados, con el fin de contar con una herramienta integral de seguimiento y evaluación del cumplimiento del derecho fundamental a la salud.

En esta misma línea, la Ley Estatutaria de Salud establece que los indicadores de goce efectivo del derecho a la salud deben servir como insumo para el diseño y la implementación de políticas públicas orientadas al mejoramiento de las condiciones de salud de la población. No obstante, esta medición no ha sido implementada de manera efectiva. Actualmente, dentro del seguimiento que se realiza en el país en materia de salud, no existen indicadores sistemáticos que permitan evaluar de forma integral y continua los avances en la garantía del derecho fundamental a la salud.

Ante esta carencia, se hizo una revisión de literatura sobre el seguimiento a la Sentencia T-760 y su relación con la materialización del goce efectivo del derecho a la salud. Esta búsqueda permitió identificar tres temáticas de análisis: i) judicialización de la política, tomando como caso concreto la Sentencia T-760 y la garantía del derecho a la salud (33-35), ii) el cumplimiento de las órdenes desde la participación ciudadana en todo el proceso de materialización de la sentencia (36), y iii) alternativas para cumplir con el mandato de la Corte Constitucional (37). Sin embargo, en ninguno de estos enfoques se establece una relación directa y cuantificable entre el cumplimiento de las órdenes y el avance real en el goce efectivo del derecho a la salud.

En cuanto a la medición, son escasos los estudios que evalúan el goce efectivo de este derecho a partir de datos objetivos y metodologías estructuradas. Predominan, por el contrario, análisis que señalan la vulneración del derecho a la salud sin respaldo estadístico. A nivel internacional, se destaca el estudio de Mitchel et al. (38), quienes evaluaron el cumplimiento del Gobierno de Nueva Zelanda frente a sus obligaciones derivadas del Pacto Internacional de Derechos Económicos, Sociales y Culturales (PIDESC). Para ello, analizaron indicadores estructurales, de proceso y de resultado en relación con los derechos a la vivienda adecuada y a la atención en salud, constituyendo una metodología que podría adaptarse al contexto colombiano.

En el ámbito nacional, algunos esfuerzos académicos e institucionales han intentado medir el goce efectivo del derecho a la salud. Uno de los casos más destacados es el estudio de Gómez (39), titulado "Goce efectivo del derecho a la salud de niños, niñas y adolescentes en Bogotá (2000-2010)". En este artículo, el autor analiza la correspondencia, la disparidad o el grado de armonía entre las políticas públicas de salud dirigidas a la infancia en Bogotá y el reconocimiento del derecho fundamental a la salud. Como parte de su metodología, Gómez adoptó una serie de indicadores para evaluar el grado de avance o retroceso en aspectos como el estado de salud, los resultados sanitarios y las inequidades, contrastándolos con los principios establecidos por la Corte Constitucional sobre el derecho a la salud.

Por su parte, la Defensoría del Pueblo (40) llevó a cabo un ejercicio de monitoreo del goce efectivo del derecho fundamental a la salud, centrado en la prestación de servicios en urgencias. Este análisis se realizó en el marco de la Observación General N.o 14 del Comité de Derechos Económicos, Sociales y Culturales de la ONU, así como de la Ley Estatutaria 1751 de 2015. El enfoque de la Defensoría incluyó la evaluación de la accesibilidad, la disponibilidad, la aceptabilidad y la calidad de los servicios de urgencias, aportando una aproximación empírica al seguimiento de este derecho desde la perspectiva de los usuarios del sistema.

A pesar de los avances normativos y jurisprudenciales orientados a garantizar el goce efectivo del derecho a la salud en Colombia, persiste una brecha significativa entre las disposiciones legales y su implementación práctica. La falta de indicadores sistemáticos, confiables y articulados que evalúen de manera integral el cumplimiento de este derecho, limita no solo el seguimiento efectivo de las órdenes de la Corte Constitucional, sino también el diseño de políticas públicas basadas en evidencia. Solo a través de la consolidación de sistemas de información robustos y de la incorporación de indicadores de goce efectivo en la evaluación de la gestión del sistema de salud, será posible avanzar hacia una garantía real, equitativa y sostenible de este derecho fundamental para toda la población.

## DISCUSIÓN

A partir de la Sentencia T-760 se evidencian una serie de transformaciones en el sistema de salud que, directa o indirectamente, parecen responder a las órdenes impartidas por la Corte y que habrían favorecido el goce efectivo del derecho a la salud. Entre los principales avances se destaca la declaración de la salud como un derecho fundamental autónomo, posteriormente regulado mediante la Ley Estatutaria 1751 de 2015. También se unificó el PBS con iguales condiciones para todos los afiliados, sin distinción entre regímenes, aunque aún está pendiente la unificación del valor de la UPC. Adicionalmente, se estableció un mecanismo formal para el acceso a medicamentos no incluidos en el PBS, lo que ha permitido garantizar tratamientos esenciales inicialmente excluidos. Finalmente, se han impulsado estrategias para identificar nuevas fuentes de financiamiento, con el objetivo de preservar el equilibrio financiero del sistema.

Ahora bien, tras más de quince años de emisión de la Sentencia T-760, el sistema de salud continúa enfrentando limitaciones, especialmente en términos de sostenibilidad financiera y acceso a los servicios de salud, lo que no permite el goce pleno del derecho a la salud. Solamente 2 de las 16 órdenes generales de la Corte han sido cumplidas plenamente y la mayoría presentan avances parciales, sin consolidarse un cumplimiento integral y sostenido. El nivel de cumplimiento general no supera una calificación media. Persisten deficiencias en las órdenes relacionadas con el acceso, el financiamiento y el flujo de recursos, incluso con algunos retrocesos particularmente en cuanto al financiamiento.

La unificación del PBS ha sido un avance significativo hacia la equidad en el sistema de salud. Sin embargo, su efectividad se ve limitada por la insuficiencia de la UPC, la persistencia de vacíos normativos y técnicos, y las barreras en el acceso a servicios. Aunque Colombia ha alcanzado una cobertura de afiliación superior al 98%, esta cifra no se traduce automáticamente en un acceso equitativo y oportuno a los servicios de salud. Las barreras persisten, especialmente para poblaciones rurales y vulnerables.

La sostenibilidad financiera sigue siendo un reto para el sistema de salud, que parece acentuarse en los últimos años. La permanencia de recobros, la siniestralidad no prevista -especialmente a partir de la pandemia del CO-VID-19-, los retrasos en pagos y la falta de transparencia en la deuda entre EPS e IPS siguen afectando la eficiencia y el funcionamiento del sistema.

La tutela continúa siendo una herramienta ampliamente utilizada por los ciudadanos para hacer efectivo su derecho a la salud, lo que evidencia una falla sistemática en la garantía de este derecho. La persistencia de las tutelas puede estar relacionada con la Ley Estatutaria de salud, la cual relaja los límites de los servicios y medicamentos del PBS; el aumento de los beneficios en salud, supeditado a una UPC de la cual se discute y cuestiona su suficiencia, y el deterioro del sistema, enmarcado últimamente por una fuerte incertidumbre.

En este contexto, la Corte Constitucional ha asumido un papel protagónico en la garantía de los derechos económicos, sociales y culturales. En el ámbito de la salud, ha buscado corregir las fallas regulatorias del sistema, ante la inacción o insuficiencia de medidas por parte del Gobierno. La intervención de la Corte evidencia la limitada implementación de las políticas públicas de salud, el bajo desempeño del sistema, la ausencia de mecanismos efectivos de supervisión, vigilancia y control, así como los problemas estructurales relacionados con la corrupción y el manejo inadecuado de los recursos del sector salud 41,42.

Pese a los avances normativos impulsados por la Corte Constitucional y la Ley Estatutaria de Salud, el país aún enfrenta una brecha considerable entre el reconocimiento legal del derecho a la salud y su goce efectivo. La ausencia de indicadores sistemáticos y aplicables a todos los niveles del sistema impide evaluar con precisión los avances en términos de disponibilidad, accesibilidad, aceptabilidad y calidad de los servicios de salud, tal como lo establece la Observación General N.° 14 de la ONU. Esta falta de medición limita el seguimiento a las órdenes judiciales y obstaculiza el diseño de políticas públicas basadas en la evidencia, manteniendo rezagos estructurales en la garantía real del derecho fundamental a la salud.

Si bien en la Sentencia se determinó que los problemas del sistema radicaban principalmente en el no cumplimiento de la normatividad en salud, y específicamente en lo contemplado en la Ley 100 de 1993, es necesario verificar que dichas órdenes resultan pertinentes para superar las fallas identificadas desde 2008 o, por el contrario, la Corte Constitucional no tomó las medidas suficientes o adecuadas, pues es claro que persisten las fallas estructurales que dieron origen a la sentencia ♥
